# Behavioral effects of a low molecular weight peptide fraction from *Phaseolus vulgaris* in rats

**DOI:** 10.1590/1414-431X2022e12314

**Published:** 2022-12-02

**Authors:** D. Graziani, J.V.V. Ribeiro, L.C. Turones, E.A. Costa, L.L. Reis-Silva, E.G. Araújo, L.G.F. de Paula, M.D. Ferreira-Junior, R.M. Gomes, H.M. Campos, P.C. Ghedini, K.A. Batista, K.F. Fernandes, C.H. Xavier

**Affiliations:** 1 Laboratório de Neurobiologia de Sistemas, Instituto de Ciências Biológicas, Universidade Federal de Goiás, Goiânia, GO, Brasil; 2 Laboratório Multiusuário de Análise de Moléculas, Células e Tecidos, Escola de Veterinária e Zootecnia, Universidade Federal de Goiás, Goiânia, GO, Brasil; 3 Laboratório de Farmacologia de Produtos Naturais e Sintéticos, Instituto de Ciências Biológicas, Universidade Federal de Goiás, Goiânia, GO, Brasil; 4 Laboratório de Fisiologia Endócrina e Metabolismo, Instituto de Ciências Biológicas, Universidade Federal de Goiás, Goiânia, GO, Brasil; 5 Laboratório de Farmacologia Bioquímica e Molecular, Instituto de Ciências Biológicas, Universidade Federal de Goiás, Goiânia, GO, Brasil; 6 Instituto Federal de Educação, Ciência e Tecnologia de Goiás, Campus Goiânia Oeste, Goiânia, GO, Brasil; 7 Laboratório de Química de Polímeros, Instituto de Ciências Biológicas, Universidade Federal de Goiás, Goiânia, GO, Brasil

**Keywords:** Bean, Phaseolus vulgaris, Peptides, Anxiety, Depression, Nutraceutical

## Abstract

Seminal studies stated that bean proteins are efficient neuronal tracers with affinity for brain tissue. A low molecular weight peptide fraction (<3kDa) from *Phaseolus vulgaris* (PV3) was previously reported to be antioxidant, non-cytotoxic, and capable of reducing reactive oxygen species and increasing nitric oxide in cells. We evaluated the effects of PV3 (5, 50, 100, 500, and 5000 µg/kg) on behavior and the molecular routes potentially involved. Acute and chronic PV3 treatments were performed before testing Wistar rats: i) in the elevated plus-maze (EPM) to assess the anxiolytic-like effect; ii) in the open field (OF) to evaluate locomotion and exploration; and iii) for depression-like behavior in forced swimming (FS). Catecholaminergic involvement was tested using the tyrosine hydroxylases (TH) enzyme inhibitor, α-methyl-DL-tyrosine (AMPT). Brain areas of chronically treated groups were dissected to assess: i) lipid peroxidation (LPO); ii) carbonylated proteins (CP); iii) superoxide dismutase (SOD) and catalase (CAT) enzymatic activities. Neuronal nitric oxide synthases (nNOS) and argininosuccinate synthase (ASS) protein expression was evaluated by western blotting. Acute treatment with PV3 increased the frequency and time spent in the EPM open arms, suggesting anxiolysis. PV3 increased crossing episodes in the OF. These PV3 effects on anxiety and locomotion were absent in the chronically treated group. Acute and chronic PV3 treatments reduced the immobility time in the FS test, suggesting an antidepressant effect. TH inhibition by AMPT reverted acute PV3 effects. PV3 decreased LPO and CP levels and SOD and CAT activities, whereas nNOS and ASS were reduced in few brain areas. In conclusion, PV3 displayed central antioxidant actions that are concomitant to catecholaminergic-dependent anxiolytic and antidepressant effects.

## Introduction

It is estimated that 100 to 500 K tons of beans are discarded annually and no longer used as human food due to hardening. Inadequate storage may damage the structure of beans, as a result of morphophysiological changes that harden the grains (1). These chemical and physical changes are known as the hard-to-cook (HTC) effect, which occurs when beans cannot soften because of insufficient water absorption during cooking (2). Therefore, efforts should be made to avoid wasting nutrients that are still present in HTC beans and to assess their nutraceutical potential. Despite possible losses in bioavailability of some nutrients, bioproducts from hardened grains have similar characteristics to non-HTC with regard to the amount of proteins, carbohydrates, lipids, vitamins, and minerals (3). In addition to the nutritional facts, these specific protein fragments may compose food-derived preparations that are beneficial to the human body (3).

In a previous study, we showed that a low molecular weight peptide fraction (<3kDa) from HTC *Phaseolus vulgaris* (PV3), the common bean, displays antioxidant effects in endothelial cells. Furthermore, the PV3, obtained from a product of low commercial value, was able to induce nitric oxide (NO) release and vasorelaxation without causing cell death and was capable of protecting endothelial cells from hydrogen peroxide-induced oxidative stress death (4). These effects suggested that PV3 might act as an antioxidant *in vivo*.

In the 1970's decade, pioneer studies found that *Phaseolus vulgaris* leucoagglutinin (PHA-L) is an efficient anterograde neuronal tracer. This groundbreaking finding resulted in more than 1000 published studies (5), paving the way for neuroscience experiments ranging from bench to bedside. This lectin has binding affinity for the neuronal membrane through an oligosaccharide complex containing galactose, N-acetylglucosamine, and mannose (5), thus revealing the probable mechanism underlying the tropism of this bean protein for neural tissue.

Some features suggest that small molecules may cross the blood-brain barrier. Lipid-mediated free diffusion, a molecular weight smaller than 400 Da, and a chemical structure with fewer than 8 hydrogen bonds are required to increase the chances of reaching the central nervous system by going through the neurovascular contact (5). Therefore, the separation of small molecules, as performed to obtain PV3, can increase the chances of penetrating cell membranes and reaching the brain areas controlling behavior. PV3 peptides with an average mass of 1.14 kDa (4) reached the cytoplasm of enterocytes and were found in the bloodstream following oral administration (6). In addition, sequencing and computational techniques allowed verifying psychopharmacological activities for PV3 compounds (4). In light of these findings, it is plausible to hypothesize that this low-molecular-weight protein fraction from hardened *Phaseolus vulgaris* would reach the central nervous system and modulate behaviors such as those related to anxiety and depression. In the present work, we assessed whether PV3 is able to affect the behavior of rats and what mechanisms are potentially involved.

## Material and Methods

All experiments were approved in July 2018 by the Federal University of Goiás (Brazil) Animal Welfare Committee (CEUA - UFG; protocol 084/2018) and were performed in accordance with the U.S. National Institutes of Health Guide for the Care and Use of Laboratory Animals and with the Brazilian Federal Law 11.794. We made all efforts to minimize the number of animals used.

### Obtaining PV3 extracts

PV3 is a peptide fraction of less than 3 kDa from hardened common bean residue of the species *Phaseolus vulgaris*, cultivar Pontal, obtained as previously described by Graziani et al. (4).

### Animals

Male Wistar rats (weighing 300-380 g) bred at the Central Animal Facility of the Federal University of Goiás (Brazil) were obtained. The animals were randomly allocated to cages of polypropylene (47×31×16 cm), each with five rats, with water and food *ad libitum* in controlled temperature and 12 h light/dark cycle.

### Drugs and reagents

All drugs were diluted in sterile saline (0.9% NaCl). The drugs tested were: i) PV3 at doses of 5, 50, 100, 500, and 5000 µg/kg; ii) the benzodiazepine diazepam (Sigma, USA) at 2 mg/kg, used as positive control for anxiety and locomotion tests; iii) the tricyclic antidepressant imipramine (Sigma) at 15 mg/kg used as positive control in depression tests; and iv) the inhibitor of tyrosine hydroxylase α-methyl-DL-tyrosine (AMPT) (Sigma) at 200 mg/kg, used to reveal the contribution of catecholaminergic pathways. All drugs and vehicle were injected by the *ip* route.

### Behavioral tests

Methods to test behavior were performed as previously described (7). All experiments were performed in a quiet and dimly illuminated room. On the experimental day, rats were taken to the laboratory for 1 h (ambientation period). Rats then received vehicle or drugs according to the experiments. In the acute trials, animals received *ip* injections (100 µL) of PV3 doses 1 h before trials. To study the effect of chronic treatment with PV3, the animals received daily injections of PV3 (50 µg/kg) or vehicle in a volume of 100 µL/day for 17 days. Details on treatments, doses, and sample size are shown in Table 1.

**Table 1:** Treatments used to assess the effects of *Phaseolus vulgaris* peptide (PV3) on different behaviors.

Treatment	Number of animals for each experiment		
	EPM	OF	FST
Acute vehicle	11	11	13
DZP 2 mg/kg	8	8	NA
Acute PV3 5 µg/kg	8	9	-
Acute PV3 50 µg/kg	14	11	18
Acute PV3 100 µg/kg	6	6	-
Acute PV3 500 µg/kg	7	6	-
Acute PV3 5000 µg/kg	6	7	-
Acute AMPT 200 mg/kg	11	10	6
AMPT 200 mg/kg + PV3 50 µg/kg	6	6	8
Imipramine 15 mg/kg	NA	NA	6
Chronic vehicle	11	10	6
Chronic PV3 50 µg/kg	12	9	8

EPM: elevated plus maze; OF: open field; FST: forced swim test; DZP: diazepam; AMPT: alpha-methyl-p-tyrosine; NA: not applicable.

#### Elevated plus maze (EPM) test - anxiety-like behavior

The anxiety-like behavior was assessed using the EPM test. Each animal underwent only one trial. The rat was placed in the center of the maze facing one of the closed arms and allowed to explore the maze for 5 min. Between experiments, the maze was cleaned with 10% alcohol to prevent olfactory clues. Six variables were measured: i) time spent in the open arms; ii) number of entries into the open arms; iii) time spent in the closed arms (with all four paws); iv) number of entries into the closed arms; v) time spent in central platform; and vi) number of total entries. A longer time and more entries in open arms, compared to vehicle, indicated anxiolysis. In addition, the number of entries into the closed arms and the total number of entries also indicated general activity or locomotor activity, which was confirmed in the open field.

#### Open field (OF) test - locomotion and exploration

The OF tests were performed just after EPM testing. Each rat was placed in the center of the field and allowed to explore the apparatus for 5 min. Between trials, the field was cleaned with 10% alcohol between experiments to prevent olfactory clues. Six variables were measured: i) time spent in the periphery; ii) time spent in the center; iii) immobility time; iv) number of crossings (number of square crossings); v) number of standing episodes (when rats were on their hind legs); vi) number of self-groomings (paw licking, nose/face grooming, and head cleaning). Immobility, crossings and self-grooming were locomotor outcomes and rearing episodes were taken as exploratory behavior.

#### Forced swim (FS) test - depression-like behavior

The depression-like behavior was assessed using the FS test. On the first day, rats were placed in a cylinder of polyvinyl chloride (24 cm in diameter and 60 cm high) filled with water (42 cm deep at 25±1°C) and allowed to swim for 15 min. Then, the rats were dried and placed in a cage warmed to approximately 38°C. Subsequently, the first drug administration (24 h before FS test) was performed. On the second day, the second and third drug administrations were performed, 5 and 1 h before the FS test, respectively. During the test, each rat was placed inside the cylinder and allowed to swim for 6 min, and the test was recorded with a video camera fixed above the pool for posterior analysis. Between experiments, the cylinder was cleaned with 10% alcohol and the water replaced to prevent olfactory clues. The immobility time (when the rat stops struggling and floats on the water, making only the necessary movements to keep its head above the water surface) was a measure of depression-like behavior.

### Biochemical analysis

The animals were divided into two groups, the control group that received only vehicle and the treatment group that received PV3 at the dose of 50 µg/kg. Thirty minutes after treatment, the animals were euthanized. The encephalon was removed and dissected into cortex, hippocampus, and striatum. Cerebral tissue samples were homogenized in 0.1 mol/L potassium phosphate buffer (KPB), pH=7.4 in a 1:6 (w/v) ratio. The homogenate was then centrifuged at 8000 *g* for 10 min at room temperature to yield the low-speed supernatant (S1) fractions, and both fractions were used in the biochemical assays.

#### Lipid peroxidation levels (LPO)

To evaluate LPO levels, the thiobarbituric acid reactive substances (TBARS) method was used. The estimation of LPO levels was performed spectrophotometrically, following the method described by Ohkawa et al. (8), with some modifications. The homogenate fractions of the cortex, hippocampus, and striatum samples were incubated with thiobarbituric acid, trichloroacetic acid (pH 3.4), and dodecyl sulfate sodium (SDS) at 95°C for 60 min. The reaction product was determined at 532 nm. For the interpretation of the results, an malondialdehyde (MDA) curve was performed, and the data are reported as equivalents of MDA in nmol/mg protein.

#### Carbonylated protein levels (CP)

CP derivatives were measured following the method described by Colombo et al. (9), with some modifications. The homogenate fractions of the cortex, hippocampus, and striatum samples were incubated with 2,4-dinitrophenylhydrazine (DNPH), which was prepared in 2 mol/L of HCl. The mixture was kept in the dark for 1 h and vortexed every 15 min. Denaturation buffer, ethanol, and hexane were then added to each tube and the final mixture was vortexed for 40 s and then centrifuged at 3000 *g* for 10 min at room temperature. The supernatant was discarded, and the pellet was washed with ethanol-ethyl acetate (1:1 v/v) and re-suspended in denaturation buffer. The sample was vortexed for 5 min and used to measure in the absorbance at 370 nm. The results are reported as nmol of carbonylated protein/mg protein.

#### Superoxide dismutase (SOD) activity

The SOD activity was spectrophotometrically determined according to the method of Misra and Fridovich (10), with some modifications. The principle of this method is the ability of the SOD enzyme to inhibit the autoxidation of epinephrine. The S1 of the cortex, hippocampus, and striatum samples were incubated with 60 mmol/L epinephrine bitartrate, and the reaction color intensity was measured at 480 nm. The enzymatic activity is reported in units (U) of SOD/mg of protein.

#### Catalase (CAT) activity

The CAT activity was determined spectrophotometrically by the H_2_O_2_ decomposition at 240 nm according to the method described by Aebi (11), with some modifications. The S1 fractions of the cortex, hippocampus, and striatum samples were incubated with 86 mmol/L of H_2_O_2_ and sodium phosphate buffer (pH 7.0). The enzymatic activity is reported in U of CAT/mg of protein.

#### Protein content

Total protein content in the cortex, hippocampus, and striatum samples was measured by the method described by Bradford (12), with some modifications. The concentration was calculated using serum bovine albumin (BSA) as the standard.

### Western blotting

These evaluations aimed at comparing the levels of neuronal nitric oxide synthases (nNOS) and argininosuccinate synthase (ASS) protein expression in encephalic areas of rats chronically injected with PV3 (50 µg/kg) or vehicle during 17 days. At the end of this chronic treatment, the animals were euthanized and their brains were extracted. Amygdala (AM), insular cortex (IC), prefrontal cortex (PfC), hippocampus (HC), hypophysis (HP), hypothalamus (HT), midbrain (MB), and medulla (M) were separated in tubes for processing. All proteins were normalized using the constitutional protein β-actin as reference. The mean of each region was compared with the mean of the control group and is reported as % of the control. The antibodies used were: anti-nNOS (mouse monoclonal; dilution: 1:500); anti-ASS (mouse monoclonal; dilution: 1:1.000); anti-β-actine (mouse monoclonal; dilution: 1:1.000), and anti-mouse IgG-HRP (rabbit; dilution: 1:1.000) (Santa Cruz Biotechnology, USA).

### Statistical analysis

The data are reported as means±SE and were analyzed using one-way ANOVA, followed by Fisher's post-test for each analysis with more than two means and the Student's *t*-test for comparison of two means. All analyses were performed using Graph Prism 8.0 software (USA). Results with P<0.05 were considered significant.

## Results

### Effects of acute PV3 injection on anxiety-like behavior

**Figure 1:**
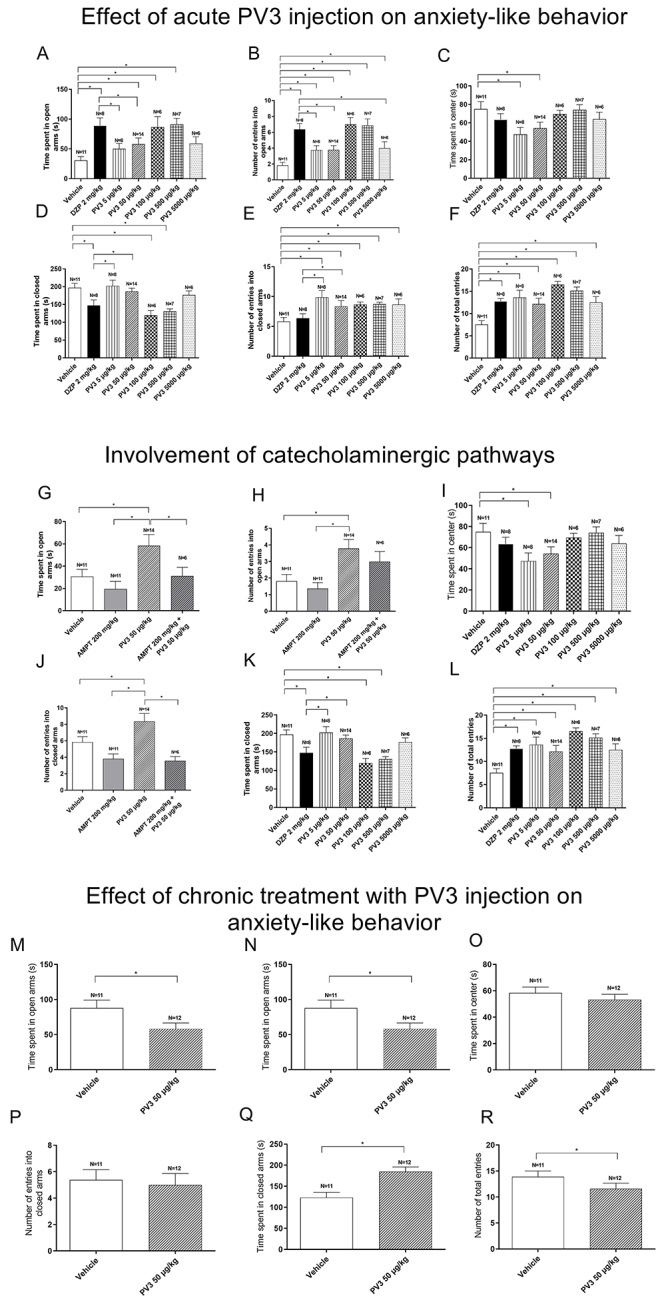
Effect of acute *Phaseolus vulgaris* peptide (PV3) injection on anxiety-like behavior of rats exposed to the elevated plus maze (panels **A**-**F**). Involvement of catecholaminergic pathways (panels **G**-**L**). Effect of the chronic treatment with PV3 (panels **M**-**R**). *P<0.05 (ANOVA or *t*-test).

Figure 1 (panels A-F) shows the parameters sampled from EPM experiments in rats injected with vehicle, diazepam, or PV3 in different doses. As expected, animals treated with the anxiolytic diazepam spent more time in the open arms compared to vehicle. Treatment with PV3 at doses of 50, 100, and 500 µg/kg increased the time in the open arms. All PV3 doses increased the frequency in open arms compared to vehicle. These findings suggested that PV3 led to anxiolysis.

PV3 was also able to increase the frequency of entries in the closed arms. PV3 100 and 500 µg/kg reduced the time spent in the closed arms compared to the vehicle group, which may reflect the increase in total entries (Table 2). All groups treated with PV3 had a higher number of total entries. Animals treated with PV3 at doses of 5 and 50 µg/kg spent less time in the center.

**Table 2:** Summary of the parameters analyzed in the elevated plus maze experiments.

Treatment	Sample (n)	Time spent in the open arms	Number of entries into the open arms	Time spent in the closed arms	Number of entries into the closed arms	Number of total entries	Time spent in the maze center
Vehicle	11	30.7±6.4	1.8±0.4	197.0±12.7	5.8±0.7	7.5±0.9	75.0±8.0
DZP 2 mg/kg	8	89.0±13.0	6.4±0.7	147.4±15.4	6.4±0.8	12.8±0.6	63.5±6.5
PV3 5 µg/kg	8	50.2±9.0	3.8±0.6	202.4±15.9	9.9±1.1	13.6±1.6	47.4±7.6
PV3 50 µg/kg	14	58.3±10.1	3.8±0.5	186.9±8.9	8.4±1.0	12.1±1.3	54.3±6.4
PV3 100 µg/kg	6	86.5±17.6	7.0±0.9	119.7±13.1	8.7±0.4	16.5±0.8	69.5±4.2
PV3 500 µg/kg	7	91.1±10.3	6.9±0.8	130.6±7.0	8.7±0.4	15.1±0.8	74.1±5.6
PV3 5000 µg/kg	6	59.1±11.2	4.0±0.8	176.9±11.5	8.7±1.0	12.5±1.3	64.1±7.5
AMPT 200 mg/kg	11	19.6±6.7	1.4±0.4	258.5±28.6	3.8±0.6	5.2±0.7	46.5±31.7
AMPT 200 mg/kg + PV3 50 µg/kg	6	31.2±7.8	3.0±0.6	237.4±10.5	3.6±0.5	7.3±0.7	5.4±3.7
Chronic vehicle	11	88.0±11.3	5.4±0.8	123.3±12.3	5.4±0.8	13.9±1.1	58.4±4.4
Chronic PV3 50 µg/kg	12	58.0±8.6	4.0±0.7	184.7±11.1	5.0±0.9	11.6±1.1	53.3±4.1

Data are reported as mean±SE. DZP: Diazepam; PV3: *Phaseolus vulgaris* peptide; AMPT: alpha-methyl-p-tyrosine.

### Catecholaminergic pathways in anxiety-like behavior

Figure 1 (panels G-L) shows the results from the EPM experiments in rats injected with vehicle, AMPT, or PV3 (50 µg/kg). The dose of 50 µg/kg was chosen because it is an intermediate dose capable of evoking anxiolytic effects in this paradigm. AMPT 200 mg/kg alone did not affect the total frequency and time spent in open arms. AMPT was able to reduce the frequency and the time spent in the open arms in animals subsequently treated with PV3. These values were similar to vehicle and AMPT groups, thus indicating the contribution of catecholaminergic neurotransmission to the PV3-evoked anxiolysis.

Treatment with AMPT increased the time spent in closed arms. When analyzing the frequency of entries into the closed arms, there was similarity between the vehicle and AMPT. It is possible to observe that the total number of entries was comparable between the vehicle group and the group treated with AMPT 200 mg/kg + PV3 50 µg/kg. Altogether, these data highlight that PV3-evoked anxiolysis relied on tyrosine-related pathways.

### Effect of chronic PV3 treatment on anxiety-like behavior

It is worth mentioning that the chronic treatment with PV3 did not cause deaths. Figure 1 (panels M-R) shows the effects of chronic treatment with PV3 on EPM parameters. Chronic treatment with PV3 (50 µg/kg) reduced the time spent in the open arms (*vs* vehicle). Furthermore, the time spent in the closed arms was increased in the group treated with PV3 50 µg/kg. In line with these results, chronic treatment with PV3 reduced the number of total entries compared to the matched control group (Table 2).

### Effect of acute PV3 treatment on locomotion/exploration

**Figure 2:**
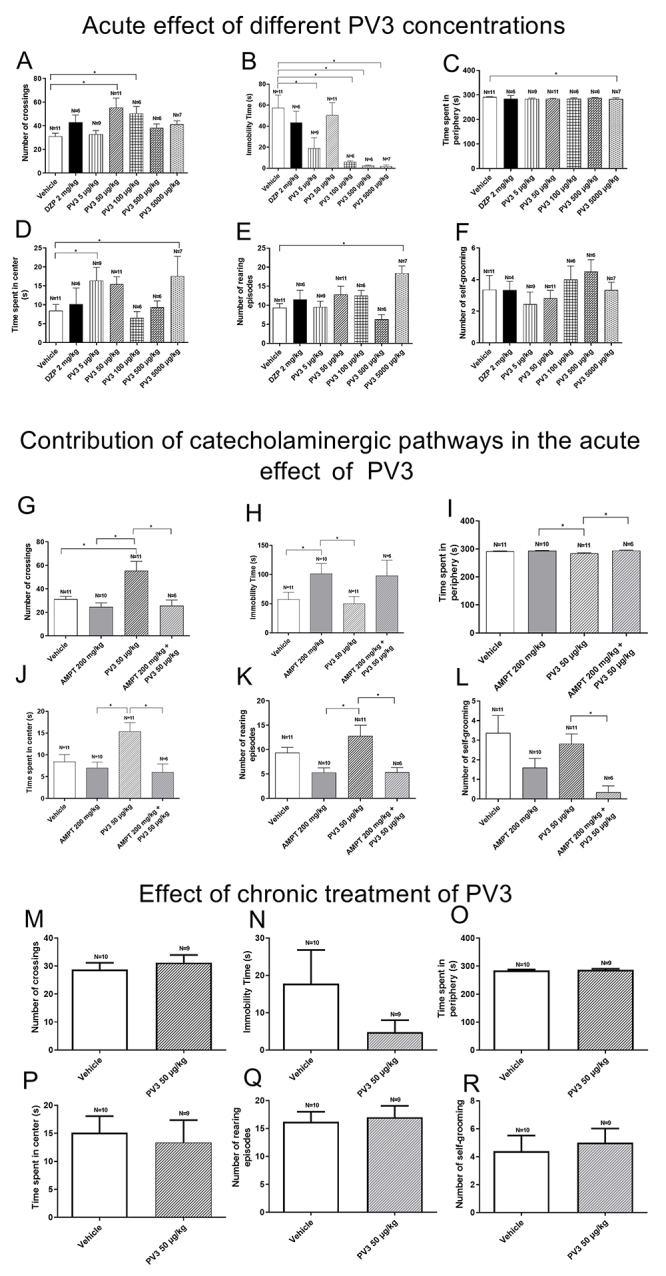
Effect of acute PV3 injection on locomotion/exploration during open field tests (**A**-**F**). Involvement of catecholaminergic pathways (**G**-**L**). Effect of chronic treatment with PV3 (**M**-**R**). Data are reported as means±SE. *P<0.05 (ANOVA or *t*-test). DZP: diazepam; AMPT: alpha-methyl-p-tyrosine.

Figure 2 (panels A-F) shows the results from OF experiments in rats injected with vehicle, diazepam, or PV3 in different doses. Crossing episodes were more frequent in animals treated with PV3 50 µg/kg and PV3 100 µg/kg, thus revealing a lack of sedative-like effects. Treatments with PV3 (5, 100, 500, and 5000 µg/kg) reduced the immobility time compared to animals treated with vehicle (Table 3). Together with the shorter immobility periods, these data suggested an anxiolytic-like effect. Furthermore, there was no difference in time spent in the periphery, demonstrating that PV3 did not promote negative influences on locomotion.

**Table 3:** Summary of parameters analyzed in the open field experiments.

Treatment	Sample(n)	Time spent in the periphery (s)	Time spent in the center (s)	Immobility time (s)	Hearing episodes	Grooming episodes	Crossing episodes
Vehicle	11	291.5±1.6	8.4±1.6	57.4±12.2	9.4±1.1	3.4±0.9	31.1±2.6
DZP 2 mg/kg	4	284.3±5.6	10.1±4.3	43.6±10.5	11.5±2.4	3.3±0.6	43.0±6.1
PV3 5 µg/kg	9	283.6±3.5	16.4±3.5	18.9±10.1	9.6±1.5	2.4±0.8	32.9±3.1
PV3 50 µg/kg	11	284.6±2.0	15.4±2.0	50.5±12.0	12.8±2.2	2.8±0.5	55.5±7.9
PV3 100 µg/kg	6	284.2±3.7	6.5±1.7	6.2±0.9	12.5±1.4	4.0±0.9	50.5±6.0
PV3 500 µg/kg	6	288.2±1.2	9.3±1.7	2.3±0.8	6.3±1.2	4.5±0.8	38.3±3.2
PV3 5.000 µg/kg	7	282.4±5.2	17.6±5.2	1.8±1.3	18.4±1.9	3.3±0.5	41.1±3.0
AMPT 200 mg/kg	10	293.0±1.3	7.0±1.3	101.8±17.1	5.3±1.0	1.6±0.5	24.7±3.4
AMPT 200 mg/kg + PV3 50 µg/kg	6	294.0±1.9	6.0±1.9	98.3±26.3	5.3±1.0	0.3±0.3	25.7±4.9
Chronic vehicle	10	284.9±3.0	15.1±3.0	17.8±9.1	16.2±1.8	4.4±1.1	28.7±2.5
Chronic PV3 50 µg/kg	9	286.7±4.0	13.4±4.0	4.8±3.2	17.0±2.0	5.0±1.0	31.2±2.7

Data are reported as mean±SE. DZP: Diazepam; PV3: *Phaseolus vulgaris* peptide; AMPT: alpha-methyl-p-tyrosine.

### Catecholaminergic pathways in the locomotor/exploratory behaviors

Figure 2 (panels G-L) shows the results from OF experiments in rats injected with vehicle, AMPT, or PV3 (50 µg/kg). Inhibition of catecholaminergic routes by AMPT increased the immobility time in the OF. These AMPT effects on immobility time were not affected by PV3 50 µg/kg. The increase in crossing episodes by PV3 50 µg/kg were reverted when the animals were pretreated with AMPT (Table 3).

### Effect of chronic PV3 treatment on locomotion/exploration

Figure 2 (panels M-R) shows the effects of chronic treatment with PV3 on OF parameters. OF parameters measured in animals chronically treated with PV3 (50 µg/kg) did not differ from the group injected with the vehicle (Table 3).

### Effect of acute and chronic PV3 treatment on depression-like behavior and the involvement of catecholaminergic pathways

**Figure 3:**
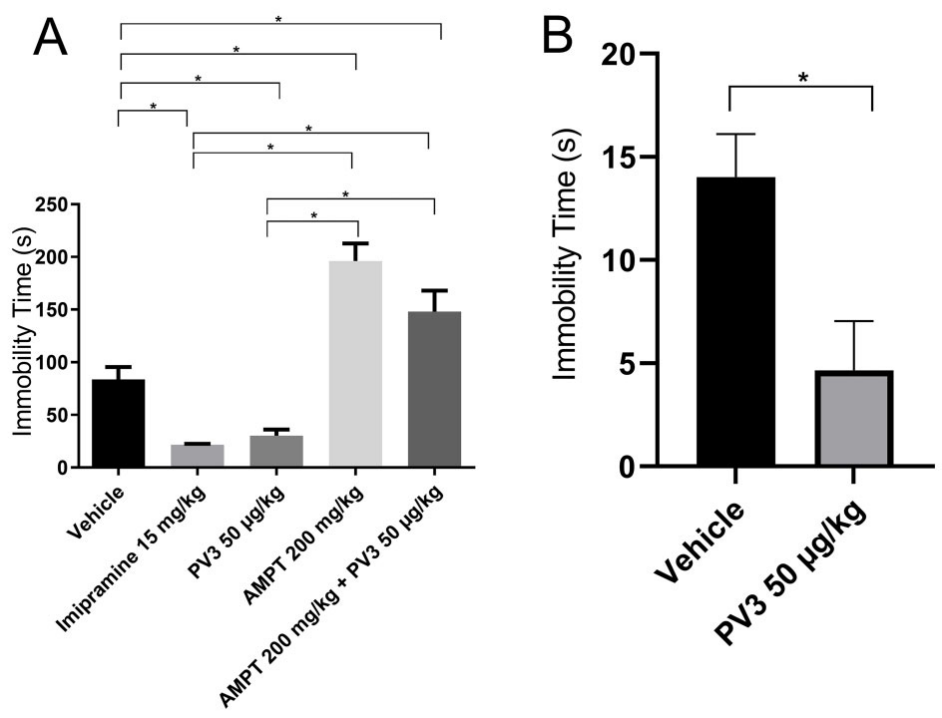
Effects of acute (**A**) and chronic (**B**) *Phaseolus vulgaris* peptide (PV3) treatments on depression-like behavior of rats exposed to the forced swim test. Data are reported as means±SE. *P<0.05 (ANOVA or *t*-test). AMPT: alpha-methyl-p-tyrosine.

Figure 3 shows the effects of acute injections of vehicle, AMPT, or PV3 on immobility time measured in forced swim tests. While PV3 50 µg/kg reduced the immobility time, this parameter was increased in animals injected with AMPT. The previous injection of AMPT reverted the PV3 effects on immobility time. The antidepressant effects caused by acute PV3 treatment were maintained in the group chronically injected with this peptide fraction (Table 4).

**Table 4:** Summary of the parameter analyzed in the forced swim experiments.

Treatment	n	Immobility time (s)
Vehicle	13	83.6±11.8
Imipramine 2 mg/kg	6	21.7±0.8
PV3 50 µg/kg	18	30.3±5.9
AMPT 200 mg/kg	6	196.2±16.5
AMPT 200 mg/kg + PV3 50 µg/kg	8	140.1±19.8
Chronic vehicle	6	14.0±2.0
Chronic PV3 50 µg/kg	8	4.6±2.4

Data are reported as mean±SE. PV3: *Phaseolus vulgaris* peptide; AMPT: alpha-methyl-p-tyrosine.

### Effect of acute and chronic PV3 treatment in antioxidant activity

**Figure 4:**
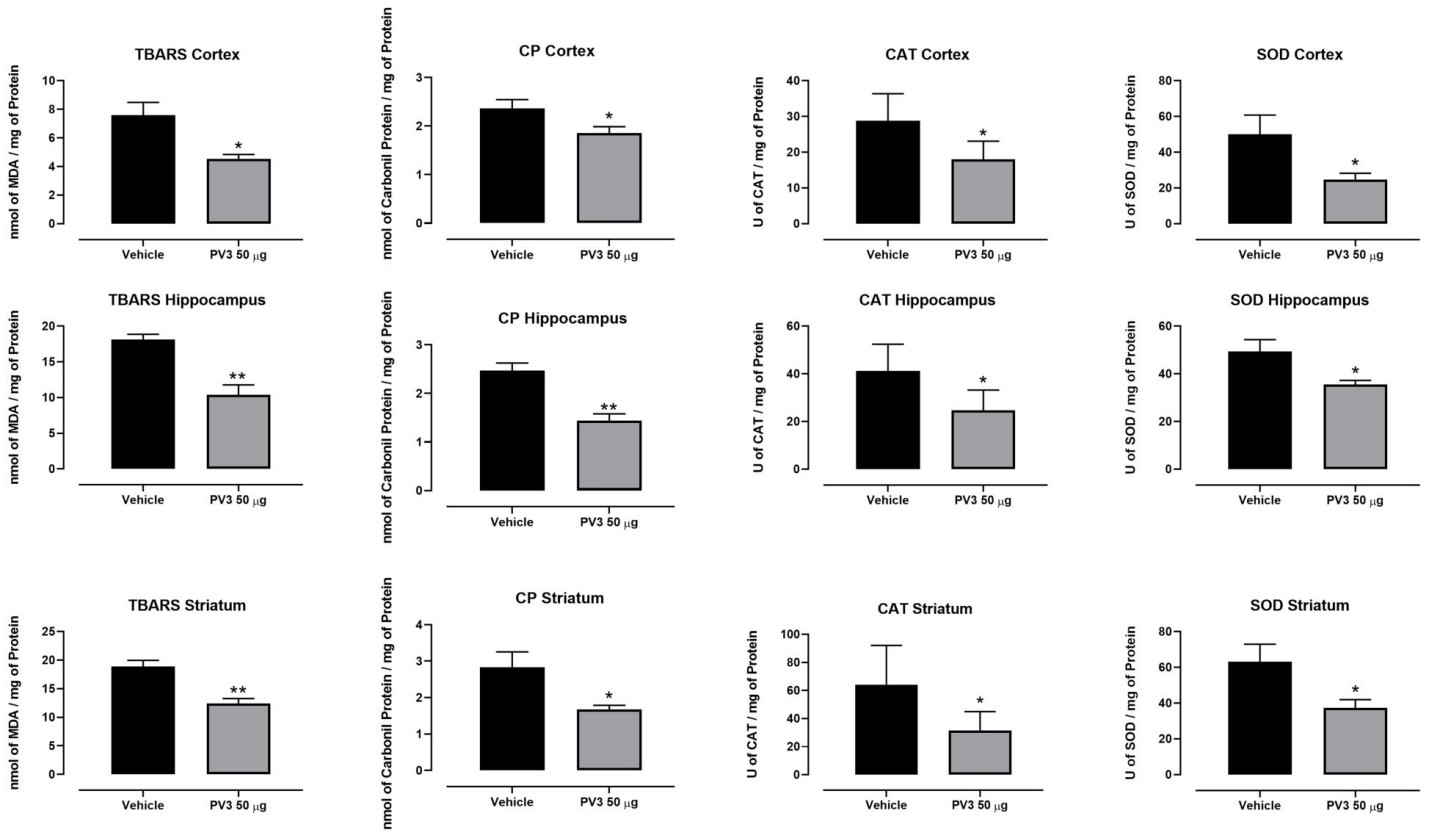
Evaluation of lipid peroxidation (TBARS), carbonylated proteins (CP), catalase (CAT), and superoxide dismutase (SOD) in the encephalic samples taken from rats acutely injected with *Phaseolus vulgaris* peptide (PV3) 50 µg/kg. Data are reported as means±SE. *P<0.05, **P<0.01 (*t*-test).

Acute injections of PV3 50 µg/kg reduced LPO and CP levels in the cortex, hippocampus, and striatum. Furthermore, the activities of SOD and CAT enzymes were reduced with the same treatment compared to the vehicle group (Figure 4).

### Levels of nNOS and ASS protein expression in encephalic areas and hypophysis

**Figure 5:**
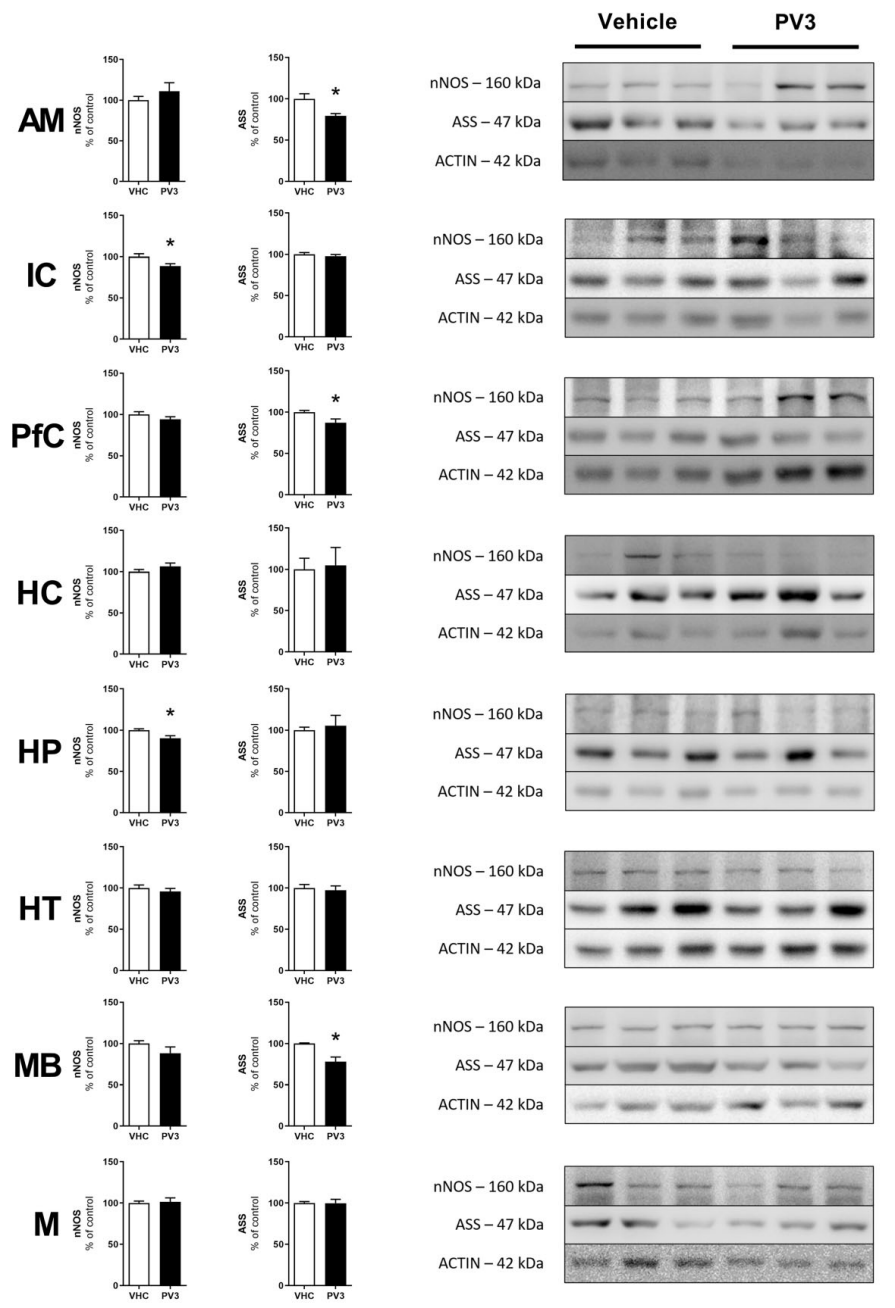
Effect of chronic treatment with *Phaseolus vulgaris* peptide (PV3) on neuronal nitric oxide synthases (nNOS) and argininosuccinate synthase (ASS) protein expression in different encephalic areas. AM: amygdala, IC: insular cortex, PfC: prefrontal cortex, HC: hippocampus, HP: hypophysis, HT: hypothalamus, MB: midbrain, and M: medulla. Data are reported as means±SE. *P<0.05 (*t*-test).

Chronic treatment with PV3 50 µg/kg provoked a reduction in the protein expression of nNOS in the pituitary and in the insular cortex. In addition, there was a downregulation of ASS in the midbrain, prefrontal cortex, and amygdala (Figure 5).

## Discussion

This study followed our prior report showing the capacity of PV3 of inducing endothelial NO release concomitantly to exerting cytoprotective and antioxidant effects (4). Our main results were: i) acute injections of PV3 produced anxiolytic- and antidepressant-like effects that rely on catecholaminergic pathways; ii) chronic treatment with PV3 displayed central antioxidant effects. PV3 was able to reduce anxiety- and depression-like behaviors without affecting locomotion. The increase in the total number of entries into the EPM arms showed that locomotion/exploration was unaffected by PV3. This was confirmed by the crossing episodes in the OF, a paradigm that is more appropriate for assessing locomotion/exploration. The longer time in the center and shorter time in the OF periphery support the EPM findings, which altogether support the conclusion that PV3 was indeed anxiolytic.

PV3 induced antioxidant effects in the hippocampus, striatum, and cortex, as revealed by the reduced LPO and CP levels in these encephalic areas. These results agreed with our previous report, in which PV3 was able to reduce the levels of reactive oxygen species (ROS) in basal conditions and during an oxidative insult induced by hydrogen peroxide exposure (4). Therefore, it is feasible to argue that PV3 central antioxidant effects may result from: i) the capacity of some PV3 components to cross the blood brain barrier; and ii) the possible change in activity and expression of some enzymes balancing the redox status within neural tissue by PV3. Intriguingly, the PV3-induced reductions in the activity of SOD and CAT enzymes suggest that this fraction promotes free radical scavenging, leading to the reduction in LPO and CP formation. It raises the possibilities that the PV3 components themselves are handling the antioxidant load and that additional targets of the antioxidant system may be involved in PV3 mechanisms of action. Due to chemical characteristics (4), such as low molecular weight and hydrophobic molecules, the hypothesis of a central antioxidant effect of PV3 becomes plausible. We believe that this phenomenon may be attributed to the antioxidant molecules present in PV3, which in hypothesis would act directly by reducing ROS rather than stimulating the activity of the aforementioned enzymes in the brain. Antioxidants have been reported to have anxiolytic and antidepressant effects by reducing encephalic ROS levels through routes other than SOD and CAT (13). Similarly, a study demonstrated that the potent antioxidant tempol was able to prevent anxiety-like behavior induced by anxiogenic drugs in rats, showing a relationship between oxidative stress and certain anxiety disorders (13).

Surprisingly, the chronic treatment with PV3 decreased the expression of nNOS in the pituitary and insular cortex and downregulated ASS in the midbrain, prefrontal cortex, and amygdala. ASS is a limiting step for NO production as it catalyzes the condensation of citrulline and aspartate to form argininosuccinate, the immediate precursor of arginine. Arginine, in turn, is the direct substrate for NO production by nNOS actions (14). In NO-producing cells, the ASS gene is expressed at low levels and pro-inflammatory signals are the main factors regulating this gene expression (15). Therefore, it is not impossible that putative inflammatory pathways are in charge of reducing the expression of nNOS and ASS in some brain areas after chronic treatment with PV3; this hypothesis, however, remains to be tested.

Besides the desirable beneficial effects, a growing body of evidence indeed points out that NO plays a pivotal role in a set of pathological conditions (14), including neurological diseases (16). NO levels in the central nervous system (CNS) are regulated by the constitutive neuronal and endothelial isoforms of the NO synthase enzyme: nNOS and eNOS. In cerebral ischemia/reperfusion injuries and in degenerative neuropathologies, there are fast glutamate-mediated increases in NO levels due to nNOS hyperactivity. During central inflammation, NO levels are strikingly high and transient, resulting from the activity of the inducible NO synthase (iNOS) isoform (for a review, see (17)). In spite of detecting downregulation in nNOS and ASS in some central areas, our results seem to diverge from any possible NO-mediated anxiogenic and depressant effects. In fact, we found that the PV3 psychopharmacological profiles were kept during chronic treatment and this does not seem to be connected to the modifications in NO-related pathways.

The lack of consistent evidence about the involvement of NO-dependent mechanisms in PV3-induced anxiolysis together with its interesting non-sedating effects that we observed raised the need for additional investigation. Therefore, we conducted complementary experiments assessing a classical neurotransmission route that is robustly involved in the organization of these behaviors: the tyrosine-derived catecholamine synthesis. Low levels of catecholaminergic neurotransmitters have been correlated to the pathogenesis of affective disorders (18). After crossing the neurovascular contact, tyrosine is used as a substrate for L-dopa formation by the action of tyrosine hydroxylase (TH) enzyme inside the brain (19). In this sense, TH inhibition with AMPT impacts the formation of dopamine (DA), epinephrine (E), and norepinephrine (NE). Although the non-classical neuropeptidergic paths (20) would aid PV3 effects, our choice was to test the contribution of classical neurotransmitters. The pharmacological strategy adopted in this study revealed that PV3 anxiolytic and antidepressant effects relied on these catecholaminergic neurotransmissions, since AMPT treatment abolished PV3 influence on the behaviors assessed in the EPM, OF, and FS tests. Nevertheless, the literature lacks reports supporting the possibility that PV3 content would directly change hormonal and neurotransmitter routes that use amino acids as substrate, such as the arginine- and tyrosine-dependent pathways.

PV3 seemed to lose its anxiolytic effect when given chronically. It is possible that the daily handling of animals, which was required for chronic treatments, modified the pattern of behavior in such a magnitude that affected EPM and OF test results. This seems plausible, since outcomes of the animals acutely injected with vehicle differed from the same injections in chronically handled control groups. Also, PV3 anxiolytic outcomes from acute treatment were comparable to those obtained from animals chronically injected with vehicle. In fact, studies show that the daily handling of animals can change behavioral and physiological responses to stress (21). Another parallel hypothesis that can be built is that chronic exposure to PV3 causes molecular adaptations, ontogenetic plasticity, or tachyphylaxis. The latter, defined as the loss of efficacy of a drug that has previously shown a response, is likely detected within the continuation phase and after the maintenance phase of treatments with centrally acting drugs such as antidepressants and anxiolytics (22). Notwithstanding, these hypotheses need further study.

It is important to remember that peptides are diverse, versatile, and low-toxicity molecules and contain short chains of amino acid residues connected by bonds that modulate a number of important cell functions (23). In the past, the search for peptides as therapeutic agents was discouraged because of their short half-life and poor oral bioavailability (24). However, more than 60 peptide drugs have been approved in major pharmaceutical markets (25). A pioneer study by our group recently reported that an oral formulation of an extract containing PV3 molecules was efficiently protected against gastric digestion, absorbed in the gut, and was found in the bloodstream (6), thus paving the way for further trials using orally administered doses of PV3.

In this work, it was possible to verify that PV3 exerted anxiolytic and antidepressant effects with catecholaminergic involvement. Furthermore, PV3 was able to reduce LPO and CP levels in the brain, extending the knowledge on the antioxidant effects previously reported in the endothelium (4). After the redox status reaches the neural tissue, it modifies innate behaviors. Jointly, the current findings led to the conclusion that PV3 can modulate centrally organized behaviors through antioxidant and catecholaminergic mechanisms. Our study is another piece of evidence on the potential nutraceutical use of hardened beans, which are usually discarded.
